# Spelling acquisition in a consistent orthography: The facilitatory effect of syllable frequency in novice spellers

**DOI:** 10.1371/journal.pone.0277700

**Published:** 2022-11-14

**Authors:** Marika Iaia, Chiara Valeria Marinelli, Francesca Vizzi, Paola Angelelli

**Affiliations:** 1 Department of Human and Social Sciences—University of Salento, Lecce, Italy; 2 Laboratory of Applied Psychology and Intervention—University of Salento, Lecce, Italy; 3 Learning Science Hub, Department of Humanities—University of Foggia, Foggia, Italy; University of Trento, ITALY

## Abstract

This study investigates the effects of two sublexical variables, syllable frequency, and word length, in the spelling acquisition of novice spellers dealing with a transparent orthography, such as Italian. Two groups of 1 st-grade Italian children were tested respectively after 4 and 8 months of schooling, with a spelling-to-dictation task of single words created ad hoc by manipulating syllable frequency orthogonally (high *vs* low frequency of the first syllable) and length (short *vs* long words). The results show that after only four months of schooling, children could offset their difficulty in writing long words by taking advantage of the high frequency of the initial syllable. However, the regularity of Italian spelling makes it easy to capture fine-sized phoneme-to-grapheme units, rendering the syllable effect no longer detectable in more schooled children.

## Introduction

Language acquisition reflects the adaptation to language regularities to which a child is exposed without any awareness or explicit intention to learn [1]. Oral and written language both have a rich statistical structure that is discovered through exposure to written texts. Even the learning of sound-to-print correspondence is probabilistic in spelling, especially in an opaque spelling language. Children who are explicitly taught the most common sound-to-spelling mappings of their writing system can use their statistical learning skills to supplement this instruction, learning about the less common mappings and about the contexts in which they occur. This sensitivity is acquired very early. Martinet et al. [2] studied French first graders after 3 months and after 9 months of literacy instruction in a spelling-to-dictation task of high- vs low-frequency irregular words and of pseudowords with lexical neighbours. Both a frequency effect in word spelling accuracy and an analogy effect in pseudoword spelling were obtained after only 3 months of reading instruction. The results suggest that from the very beginning of literacy acquisition, children acquire specific orthographic knowledge. Evidence of reliance on distributional information in novice spellers is also reported in more consistent orthographies (i.e., transparent orthography, in which an efficient phoneme-to-grapheme procedure would be sufficient in spelling most of the words) such as Italian: Angelelli et al. [3] examined word and pseudoword spelling of first, second, and fourth Italian graders. The results showed that there was already a sensitivity to probabilistic cues in spelling in first grade. High-frequency (*i*.*e*., typical) sound-spelling mappings were spelled more correctly than low-frequency (*i*.*e*., atypical) ones for both words and pseudowords. Accuracy in spelling atypical mappings emerged progressively as a function of word frequency and schooling, with greater use of atypical mappings with the progressive development of whole-word lexical representations. Children learn to extract regularity in orthography, and rely on this probabilistic information to compensate for their difficulty when lexical processing was not available, as in the case of low-frequency words, and/or in the case of dyslexic/dysgraphic children [4].

According to the Integration of Multiple Patterns (IMP [5]) model, there are multiple sources of information within the writing system that children use to increase the chances of making the right spelling choice. Children learn both probabilistic patterns and deterministic patterns. In addition, writers use multiple patterns even for the same word; the use of multiple patterns makes spelling more motivated and less arbitrary, reducing the need to rely on rote memory (the storage of shapes of letters and the spellings of words). Children tend to do well when several patterns converge toward the correct answer, while they have more difficulty when this is not the case [5]. In particular, children learn about two classes of patterns: those involving the external form of writing (what it looks like) and those involving the connections between written symbols and language, that is, the internal function of writing (how it functions to represent language). These general patterns can be of different types, including grapho-tactic, phonological, and morphological. Depending on the nature of the writing system they are learning, children also need to know that speech can be conceptualized in terms of phonemes, morphemes, words, and sentences. Limitations in language knowledge can lead to slow or incomplete learning of the patterns that link writing units to speech units [5]. Children develop some knowledge of the outward form of writing as early as 2 or 3 years old, long before they learn how written symbols are connected to language [6]. Evidence for the early use of sensitivity to various sublexical orthographic units larger than the single phoneme-grapheme correspondences has been reported in both opaque and transparent orthographies. In fact, a child might process sublexical units larger than a single grapheme, but smaller than a word, such as syllables, bigrams, and morphemes. The last one is the smallest unit of language with meaning (for example “cas-INA” for which the suffix INA indicates that the house is small). Therefore, then both lexical processing and sublexical information of graphemes co-occurrence (in the reported example the high frequency of co-occurrence of the sequence of letters INA) might produce an advantage in morpheme processing. In the cases of syllables and bigrams only sublexical information of the sequence of letters co-occurring might be used as a source of information facilitating spelling. In the case of bigrams, the frequency of co-occurring letters is independent of the position of the letters in the word (for example in the case of “casa” -house- the mean of frequency of occurrence of C-A, A-S, S-A letter is computed), while for syllable the frequency of co-occurring letters is constrained by position and the frequency of only syllable is considered (in the example reported for the syllable CA and SA).

A large set of studies examined morphological effects, in particular in pseudoword spelling. Young English- and French-speaking children showed morphological facilitation in spelling, indicating that children do not rely (at least not exclusively) on phoneme-to-grapheme mapping rules, but also learn intra- and inter-word regularities and use these chunks of information as spelling units [for a review, see 7]. Morphological facilitation in spelling was also found in orthographies with consistent phoneme-to-grapheme correspondences, such as Spanish [8] and Finnish [9]. Interestingly this sensitivity to the morphological structure was also found in very young children (by the end of the first year of schooling) [9]. A clear advantage in spelling pseudowords in Italian made up of real roots and suffixes compared to non-morphemic pseudowords was reported in both normally developing third- and fifth-grade children [10], as well as in children with dyslexia [3]. The exposure of developing readers in a very transparent orthography to these frequently occurring chunks of sound and meaning in speech, and their corresponding orthographic patterns in writing, also allows morphemes to become relatively independent spelling units, and facilitate spelling in the early phase of writing acquisition.

The second set of studies showing that spelling does not consist of producing a mere linear sequence of letters deals with sensitivity to grapho-tactic patterns of the orthography: frequent or legal letter associations or positions (*e*.*g*., [1, 11–13]). Starting from first grade, English- and French-speaking children preferred the most frequent phonological mapping in reading (e.g., [1]), and orthographic solutions in spelling (*e*.*g*., [12]). In a recent series of studies [4, 14, 15] our group also investigated whether young children, as well as children with dyslexia, in a consistent orthography such as Italian use larger sublexical units than phoneme-to-grapheme correspondences, reflecting knowledge of the distributional properties of the orthography and the frequency of co-occurring letters. In particular, in these studies, we tested spelling and the ability to write segments with typical or atypical mappings (orthographic solutions that occur more or less frequently in Italian orthography) in stimuli with unpredictable spelling. At the end of the first year of school, children were found to be more sensitive to segment frequency: they adopt spelling units bigger than the phoneme-to-grapheme units that occur more frequently in Italian orthography.

Another sublexical unit larger than the single phoneme/grapheme, but smaller than whole words, and facilitating spelling might be the syllable: two or more letters that co-occur together in an orthography. As far as we know, however, the possible role of syllable frequency in modulating spelling accuracy has not yet been studied. It is already documented that the syllable plays an important role in a series of linguistic tasks, and in several orthographies (for a review [16, 17]). Studies manipulating syllabic frequency in lexical decision tasks in Spanish [18–21] showed that words are recognized more slowly if they have a high-frequency first syllable. Carreiras and Perea [22] found that syllable frequency had a facilitatory effect in naming pseudowords: stimuli with a high-frequency first syllable were read faster than those with a low-frequency first syllable; when the high-frequency syllable was in the second position there was no effect on reading speed. As regards writing, there is growing literature demonstrating that syllables modulate the spatiotemporal aspects of handwriting movements. The syllabic structure of a word, defined as the different options of vowels and consonants organized within a syllable (*e*.*g*., CV, VC, CCV, or CVC), influences the duration of movements: inter-letter intervals are longer when a syllable boundary falls between the two letters than when the two letters belong to the same syllable [23]. The simultaneous activation of different sublexical representation levels (phoneme-to-grapheme, bigrams, syllables) leads to supplementary cognitive load, increasing movement durations. Lambert et al. [24] found that the number of syllables also affected the latencies of university students when spelling pseudowords, suggesting that when items do not activate a representation in the orthographic lexicon, the letter strings are chunked into syllables. After the students had spelled the items twice, latencies also increased as a function of the number of syllables for words. This suggests that words are also segmented into their syllable constituents after the activation of the orthographic representation. Many results, therefore, converge towards the idea that syllables are processing units in word writing production [25]. From a developmental point of view, syllables were found to mediate visual parsing and the motor programming of primary school children, showing that syllable-oriented spelling strategies are present very early in handwriting acquisition. Kandel and Valdois [26] studied French first to fifth-graders in a copying task using words and pseudowords and found that first- and second-grade children decomposed items into syllable-size units (the syllable was a functional unit for both visual parsing and motor programming), and that the syllabic structure continued to constrain motor programming in older children (when the syllable is no longer used for visual parsing). In summary, studies strongly suggest that syllables regulate the timing of handwriting programming, and this syllable-oriented writing strategy supports the idea that syllables are an essential component of orthographic representations. This is in line with neuropsychological data revealing that orthographic representation codes the grapho-syllabic boundaries in words (i.e., processing units in handwritten production that obey grapho-tactic constraints that define the combination of grapheme consonants and graphemic vowels) (*e*.*g*. [27]).

The present study investigated the role of syllable frequency and stimulus length in modulating spelling accuracy in a very early phase of spelling acquisition in a transparent orthography such as Italian. Due to the simplicity of sublexical spelling in Italian, we tested children attending first grade respectively after 4 and 8 months of schooling. We examined spelling accuracy in a spelling-to-dictation task of single words created *ad hoc* by orthogonally manipulating the syllable frequency (high- *vs* low-frequency of the first syllable) with the word length (short *vs* long). We chose to use words instead of pseudowords in order to make the task more ecologically valid and salient for such young children. We expected that the high frequency syllables would particularly assist in the spelling of long rather than short stimuli. Spelling long stimuli is more error-prone than spelling short ones, due to the heavier demands on buffering mechanisms (i.e., on phonological and graphic short-term memory) and the greater number of letters to be spelt and ortho-tactic difficulties (compared to short stimuli). The presence of a high frequency syllable assists the sublexical procedure and buffering processes, however, the high consistency of phoneme-to-grapheme correspondences in Italian means that it may be possible for very young children to promptly grasp these fine-sized spelling units, overruling the effect of syllable frequency, at least in spelling accuracy.

## Method

### Participants

A total of 100 children (55 boys, 45 girls) attending the first year of school in the south of Italy were enrolled. The first group of sixty-three participants (31 boys, 32 girls; mean age = 6.51 years, SD = 0.29) was tested in January, and the second group of thirty-six first-grade children (24 boys, 13 girls; mean age = 6.81 years, SD 0.31) was tested in May. Both groups of children attended the same primary school. All children were monolingual.

The children had to fulfil the following criteria to be included in the study: *i)* absence of certificated neurodevelopmental disorders; *ii*) normal performance (within 2 SD of the mean) on *Raven’s Coloured Progressive Matrices* (*CPM)* [according to Pruneti et al. (e.g., [28]), the cut-off score for this age level is 14, *i*.*e*. the score above the 10th percentile], and *iii*) adequate socio-educational conditions (none of the children were reported by their teachers for socio-economic disadvantage).

All children attended school normally, as the study was undertaken before the COVID-19 pandemic crisis. Written informed consent was obtained from all participants for inclusion in the study. In particular, parents were informed of the screening activities and authorized their child’s participation by signing the appropriate informed consent paperwork. The study was conducted according to the principles of the Helsinki Declaration and was approved by school authorities. The study was also approved by the Ethic committee of Psychological Research of the Department of History, Society and Human Studies -University of Salento.

### Experimental procedure and material

The experimental stimuli included 60 words, with regular transcription (one-sound-to-one letter mapping) which were subdivided as follows (see S1 Appendix):

- 15 short words with a high frequency of the first syllable;
- 15 short words with low frequency of the first syllable;
- 15 long words with a high frequency of the first syllable;
- 15 long words with low frequency of the first syllable;

Words were selected from the dictionary of written Italian frequency for children [29]. Short words were composed of 4/5 letters (mean = 4.10, SD = 0.31); long words were composed of 6/8 letters (mean = 6.83, SD = 0.79). Words were of medium-low frequency (mean of the natural logarithm of frequency = 3.15, SD = 0.92). We manipulated the frequency of occurrence of the first syllable of each word, which always corresponded to the first two letters. In fact, the syllable boundaries were always after the first bigram. High-frequency first syllables had a frequency higher than 1 (mean = 1.70, SD = 0.60), and low-frequency first syllables had a frequency lower than 0.11 (mean = 0.07, SD = 0.04).

The sets were matched for the number of letters, orthographic complexity, children’s word frequency [30], bigram frequency (according to children’s word frequency corpora) [29], the mean syllable frequency of all other syllables except the first one [30], stress position, and N-size (according to the Corpus and Frequency Lexicon of Written Italian (CoLFIS)) [31] (http://dpss.psy.unipd.it/claudio/vicini2.php).

Words were presented in random order and given to children in a spelling-to-dictation task. The experimenter read each item aloud in a neutral tone and the children were required to write each stimulus in either capital or lowercase letters (as they preferred). No feedback was provided about the accuracy of the written response. The response was considered incorrect (and so counted as 0 for the number of correct responses) if there were one or more omissions, substitutions, transpositions or insertions of letters, irrespective of typology and number of errors. The written productions that matched the target words were counted as correct. The number of correct productions was counted for every participant. Children were tested in small groups (4/5 children) in a quiet room. The experimental session lasted about 30 minutes.

### Data analysis

Accuracy percentages were analyzed by means of ANOVA with *the observational moment* (half *vs* end of the first grade) as a between-participant factor and *syllable frequency* (high *vs* low frequency), and *length* (short *vs* long words) as a within-participant factor.

## Results

The ANOVA highlighted the significance of the main effect of *observational moment* (F _(1,98)_ = 6.17, p < .05) and *length* (F _(1,98)_ = 116.71, p < .0001): accuracy was 73.2% and 62.5% respectively for more schooled and less schooled children and 76.8% and 59.0% respectively for short and long words. The syllable frequency effect was not significant (F _(1,98)_ = .54, ns; 68.3% *vs* 67.5% respectively for words with high- and low-frequency syllables), but interacted with other variables examined.

The interactions of *length* with *observational moment* (F _(1,98)_ = 14.84, p < .001), *length* with *syllable frequency* (F _(1,98)_ = 8.79, p < .01) and *length* with *syllable frequency* and *observational moment* (F _(1,98)_ = 8.27, p < .01) were significant.

The *length* by *observational moment* interaction highlighted a greater length effect among less schooled (difference between long and short words = 24.1%, p < .001) than more schooled children (difference = 11.4%, p < .001). Group differences were evident in spelling long words (50.5% and 67.5% of accuracy respectively for less and more schooled children, p < .0001), but not in spelling short words, for which groups showed a similar accuracy (74.6% and 79.0% respectively; S1 Fig).

The *length* by *syllable frequency* interaction revealed greater accuracy in spelling long words with a high-frequency first syllable compared to those beginning with low-frequency syllables (59.2%, *vs* 54.5% respectively, p < 0.01), but not in spelling short words, where first syllable frequency did not affect accuracy (74.9% and 77.7% respectively). A length effect was evident in spelling words with high-frequency first syllables (difference between long and short words = 14.8%, p < .0001), and in spelling words with low-frequency first syllables (difference between long and short words = 20.8%, p < .0001) (S2 Fig).

The *length* by *syllable frequency* by *observational moment* interaction suggested the absence of any significant effect between both groups for short words: accuracy was quite high at each observational moment (about 70%-80%), and no syllable frequency effects were significant. Accuracy was lower with longer words for less schooled children, and a first syllable frequency effect was detectable in this group: long words with high-frequency first syllables were spelled with 54.1% accuracy; long words with low-frequency first syllables were spelled with lower accuracy (46.8%; at least p < .05) (S3 Fig). Children with more schooling spelled long words with high *vs* low first syllable frequency comparably (ranging from 67% to 68%), without any significant difference in syllable frequency (S1 Table).

The data underlying the results presented in the study are available from Supporting Information files (S1 Dataset).

## Discussion

Several studies have found that the syllable is a privileged candidate as a processing unit in the production of written words. The evidence for this is mainly from studies focusing on handwriting movements [24, 26, 32, 33], which found that syllables regulate the spatiotemporal aspects of handwriting programming. We were interested to examine whether the frequency of the first syllable also has an effect on spelling accuracy, and in learners of a transparent orthography such as Italian. Previous studies on spelling acquisition in transparent orthographies (for a review see [34], for data on Italian see [35, 36]) showed a prevalent reliance on sublexical processes, but to our knowledge, this is the first study investigating the role of syllable frequency in spelling accuracy. A syllable is a sublexical unit of intermediate size, which may help novice spellers who are not yet fully efficient in fine phoneme-to-grapheme mappings, to manage the sound-to-print transcribing processes. Due to the very easy acquisition of spelling in Italian, we examined first-grade children after 4 and 8 months of schooling through a spelling-to-dictation task involving regular words varied for the frequency of the first syllable and for stimulus length.

The results deserve several comments. Firstly, they confirm the simplicity of spelling acquisition in a transparent orthography such as Italian (coherently with [36, 37]). After four months of school, children were very accurate in spelling short words, there was also a significant length effect among older children. In fact, results clearly showed that word length affected spelling accuracy, with longer words spelled more poorly than short words at both observational points. It is known, in fact, that length is a variable that makes the sublexical procedure more complex (see [38, 39]). For longer phonological strings (to be transcribed), there is a higher presence of orthotactic difficulties (e.g., doubling consonants, clusters of consonants, etc.) as well as more numerous possible error loci in the course of the two conversion processes (*i*.*e*., acoustic-to-phonological and phonological-to-orthographic) that select phonological and graphemic elements, and the heavier the demand on the two memory buffers (phonological and graphemic buffers). Interestingly, the length effect was detected in the spelling of words with regular transcription (for which a reliance on the lexical procedure, in which word-specific orthographic representations are accessed, might corroborate their spelling), thus confirming a major reliance on sublexical spelling processes, at least at the examined age. This result is coherent with Notarnicola et al. [35], who found that length was a powerful factor in modulating spelling performance in the early stages of learning, and became progressively less critical later on in the case of high-frequency words. Despite the ease of spelling acquisition through sublexical mapping in transparent orthographies, the latter process thus does not reach accuracy as early as expected (see also [36]).

Interestingly, the results provide new information about spelling acquisition: absolute beginners exploit not only phoneme-to-grapheme units but also intermediate units such as syllables, being sensitive to their frequency of occurrence. In fact, we found that syllable frequency had a facilitatory effect in very young spellers: long words starting with high-frequency syllables were spelled more accurately than those starting with low-frequency syllables. The frequency of the first syllable did not promote further spelling accuracy at the end of the first grade, regardless of the length of stimuli. It seems reasonable that younger children may overcome their difficulties in spelling long error-prone words if they can rely, at least for the first piece, on a longer processing unit, such as the syllable. As they become more efficient in fine phoneme-to-grapheme mapping, the facilitation induced by the syllable frequency is no longer detectable. Syllable frequency thus positively supports the conditions that challenge sublexical processing more, i.e. the spelling of long stimuli in absolute beginners. Syllable frequency information also does not modulate spelling accuracy with short words in very young learners, at least in a highly transparent orthography such as Italian.

Several studies show that children who have started using phonology to select letters possess some grapho-tactic knowledge (e.g., [12, 40, 41]) as well as sensitivity to distributional information (e.g., [4, 15, 16]) and use this knowledge when making their spelling choices when using the sublexical mapping. Also, intermediate sublexical units (*e*.*g*., [42]) positively affect spelling accuracy in transparent orthographies (see for example morphemes [9]).

The potential effect of syllable frequency and the simultaneous activation of this representation level has so far only been documented in studies analysing the timing of handwriting programming. For example, Kandel et al. [16] highlighted a syllable-by-syllable writing strategy in the handwriting of 3^rd^ and 4^th^ graders, although with a developmental trend indicating decreased effects in older children, for whom handwriting had become more skilled and automatic. According to these authors, young children would only have a syllable module (that encodes information on the syllable structure of words and on the position of grapho-syllabic boundaries), and the activation of the syllable units would depend on syllable frequency. As knowledge of letter co-occurrence (*i*.*e*., bigrams) and knowledge of fine-sized phonemes-to-graphemes relationships increases, the children incorporated a letter module that would overrule the effect of syllable frequency at a later stage of development.

In the present study, the first syllable coincided with the first two phonemes, and no cases were presented in which the syllable boundary fell beyond the first bigram. The data, therefore, does not allow disambiguating the differential potential effects of syllable *vs* bigram frequency on spelling accuracy. Further studies are necessary to achieve this. Our results confirm that children grasp phoneme-to-grapheme mapping as they progress through school, and as their knowledge of the relationship between phonemes and graphemes increases, and this acquisition overrides the effect of syllable frequency, at least as seen when analysing accuracy.

From a theoretical point of view, it is interesting to speculate on the possible mechanisms underlying the syllable effect on spelling accuracy observed here. On the one hand, syllable frequency may positively affect spelling accuracy by lightening the working memory load. However, Kandel et al. [32] found that the syllabic units children use in writing had an orthographic rather than phonological format. The authors used French words, for which syllabification is not the same in speech and written language. Third- to fifth-grade children wrote words that were monosyllables phonologically, but bisyllables orthographically (*e*.*g*., barque (boat), which is monosyllabic phonologically [baRk] but bisyllabic orthographically (bar.que)). These stimuli were matched to words that were bi-syllables both phonologically and orthographically (*e*.*g*., balcon (balcony), which is bisyllabic phonologically [bal.ko˜] and orthographically (bal.con)). The results indicated that both types of words were segmented according to graphosyllabic, rather than phonologic patterns (*i*.*e*., processes exclusively derived from speech production). The syllable facilitation we observed in spelling accuracy may therefore originate from accessing and retrieving pre-assembled orthographic syllable units, which may become relatively independent spelling units thanks to the ability of children to implicitly extract orthographic regularities. The activation of syllable level representation may reduce the accumulation of spelling costs and errors and assist children engaged in consolidating phoneme-to-grapheme analysis. As stated in the introduction various sources of evidence in the literature indicate that children are able to learn orthographic regularities [1, 4, 14, 15].

In conclusion, our results indicate that very young Italian children are sensitive to syllable frequency when they spell long words. Children integrate the still immature and error-prone phoneme-to-grapheme strategy with their distributional knowledge at a sublexical level: they were advantaged in spelling more frequent syllables compared to to infrequent ones. The consistency of Italian orthography makes the acquisition of fine phoneme-to-grapheme correspondence rules in spelling very easy, which means that this effect is not yet detectable at the end of the first year of schooling.

Our study is not free of limitations, and some notes of caution are needed. It is possible that the effect of syllable frequency may also be identified in more schooled children by studying the temporal parameters of writing. Kandel et al. [16] studied handwriting productions, and found a syllable effect continuing in 3^rd^ grade children, and, to a lesser extent also in 4^th^ graders. It would also be interesting to investigate whether syllable frequency continues to facilitate spelling in the case of pseudowords (a condition in which lexical support fails and there is a heavier demand on sublexical processing), and whether syllables beyond the initial one may also continue to modulate spelling performance in older children. It also seems possible that syllables with a high frequency of occurrence at the end of words might support the graphemic buffer and then facilitate spelling. This is a cross-sectional, and not a longitudinal study, and so it is possible that other intervening variables may affect the results.

Overall, the present study adds knowledge regarding the sublexical information exploited in the spelling acquisition of novice spellers dealing with a transparent orthography and provides guidance for further studies.

## Supporting information

S1 Appendix Experimental stimuli in each set. (PDF)

S1 Fig Percentages of accuracy for short vs long words in the two observational moments. T1 = less schooled first graders; T2 = more schooled first graders.
(PDF)

S2 Fig The figure reports the percentages of accuracy in short vs long word spelling as a function of syllable frequency. hf = high frequency syllable; lf = low frequency syllable.
(PDF)

S3 Fig The figure reports the percentages of accuracy in spelling long words as a function of syllable frequency in the two observational moments. T1 = less schooled first graders; T2 = more schooled; hf = high frequency syllable; lf = low frequency syllable.
(PDF)

S1 Table Mean percentage of accuracy (and Standard Error) of spelling short and long words, with high and low syllable frequency at T1 (January) and T2 (May). (PDF)

S1 Dataset Data underlying the results presented in the study. (XLS)
