# Supplementary material for: Spelling acquisition in a consistent orthography: The facilitatory effect of syllable frequency in novice spellers
Source: PLoS One. 2022 Nov 14;17(11):e0277700. doi: 10.1371/journal.pone.0277700 (PMC9662710; doi:10.1371/journal.pone.0277700)
Supplement: S1 Appendix — (PDF) [file pone.0277700.s001.pdf]

S1 Appendix: Experimental stimuli in each set.

Syllable frequency	Short words	First syllable frequency	N size	N° of letters	Word frequency	Long words	First syllable frequency	N size	N° of letters	Word frequency
hf syllable	tema	2.34	12	4	3.93	insetto	2.22	4	7	4.55
hf syllable	noce	2.14	9	4	4.01	colonna	1.19	2	7	3.78
hf syllable	rete	1.83	11	4	4.37	regina	1.83	3	6	4.38
hf syllable	dito	1.35	15	4	4.86	rispetto	2.35	3	8	4.04
hf syllable	seta	1.01	12	4	3.30	dolore	1.41	3	6	3.91
hf syllable	tifo	3.06	11	4	2.48	divano	1.35	7	6	4.28
hf syllable	misto	1.20	9	5	2.20	tinello	3.06	3	8	1.95
hf syllable	nodo	2.14	13	4	2.56	tenebra	2.34	2	7	2.71
hf syllable	remo	1.83	11	4	2.64	castoro	1.62	3	7	2.56
hf syllable	biro	0.76	10	4	2.30	libreria	1.79	4	8	2.64
hf syllable	cobra	1.19	2	5	1.79	corallo	1.19	2	7	2.56
hf syllable	dote	1.41	14	4	3.09	ritratto	2.35	3	8	2.83
hf syllable	diga	1.35	11	4	2.48	mastino	0.91	4	7	1.61
hf syllable	lido	1.79	9	4	1.95	dipinto	1.35	4	7	2.64
hf syllable	teso	2.34	11	4	2.48	narice	1.33	1	6	1.95
lf syllable	buca	0.11	14	4	3.56	effetto	0.02	2	7	4.62
lf syllable	puro	0.11	15	4	3.61	errore	0.03	3	6	4.06
lf syllable	zona	0.10	13	4	4.93	ossigeno	0.04	0	8	4.36
lf syllable	osso	0.04	8	4	4.57	ottimo	0.04	5	6	4.29
lf syllable	onda	0.03	4	4	4.48	ultimo	0.03	1	6	3.97
lf syllable	urlo	0.02	6	4	3.37	fucile	0.10	5	6	3.33
lf syllable	ruga	0.06	5	4	2.77	pulito	0.11	5	6	4.87
lf syllable	rude	0.06	9	4	1.79	urbano	0.02	6	6	2.77
lf syllable	fusa	0.10	11	4	2.40	offerta	0.03	3	7	2.77
lf syllable	fune	0.10	9	4	2.30	rubrica	0.06	0	7	2.77
lf syllable	fusto	0.10	6	5	3.00	gustoso	0.08	3	7	2.83
lf syllable	ente	0.10	4	4	2.48	funerale	0.10	1	8	2.83
lf syllable	orco	0.10	10	4	2.64	avvocato	0.10	2	8	3.00
lf syllable	orma	0.10	7	4	2.30	organo	0.10	4	6	3.00
lf syllable	puma	0.11	11	4	2.30	bufera	0.11	1	6	2.20
