# Supplementary material for: Spelling acquisition in a consistent orthography: The facilitatory effect of syllable frequency in novice spellers
Source: PLoS One. 2022 Nov 14;17(11):e0277700. doi: 10.1371/journal.pone.0277700 (PMC9662710; doi:10.1371/journal.pone.0277700)

S1 Fig. Percentages of accuracy for short vs long words in the two observational moments. T1 = less schooled first graders; T2 = more schooled first graders.

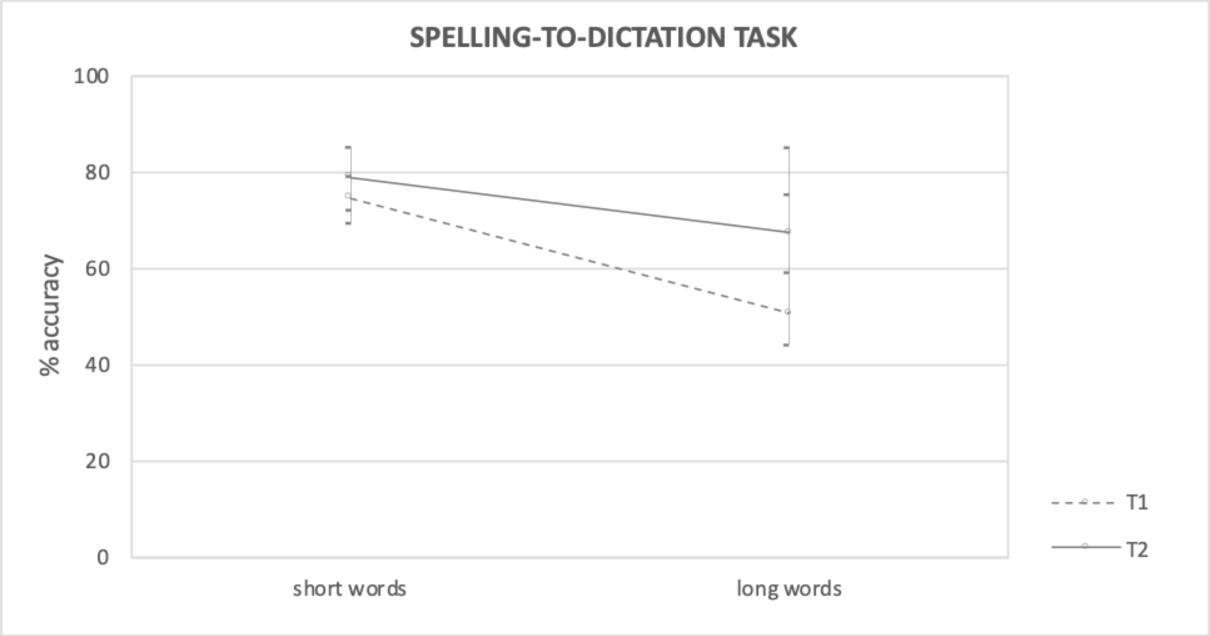

Supplement: S1 Fig — T1 = less schooled first graders; T2 = more schooled first graders. (PDF) [file pone.0277700.s002.pdf]
