# Supplementary material for: Spelling acquisition in a consistent orthography: The facilitatory effect of syllable frequency in novice spellers
Source: PLoS One. 2022 Nov 14;17(11):e0277700. doi: 10.1371/journal.pone.0277700 (PMC9662710; doi:10.1371/journal.pone.0277700)

**S2 Fig.** The figure reports the percentages of accuracy in short vs long word spelling as a function of syllable frequency. hf = high frequency syllable; lf = low frequency syllable.

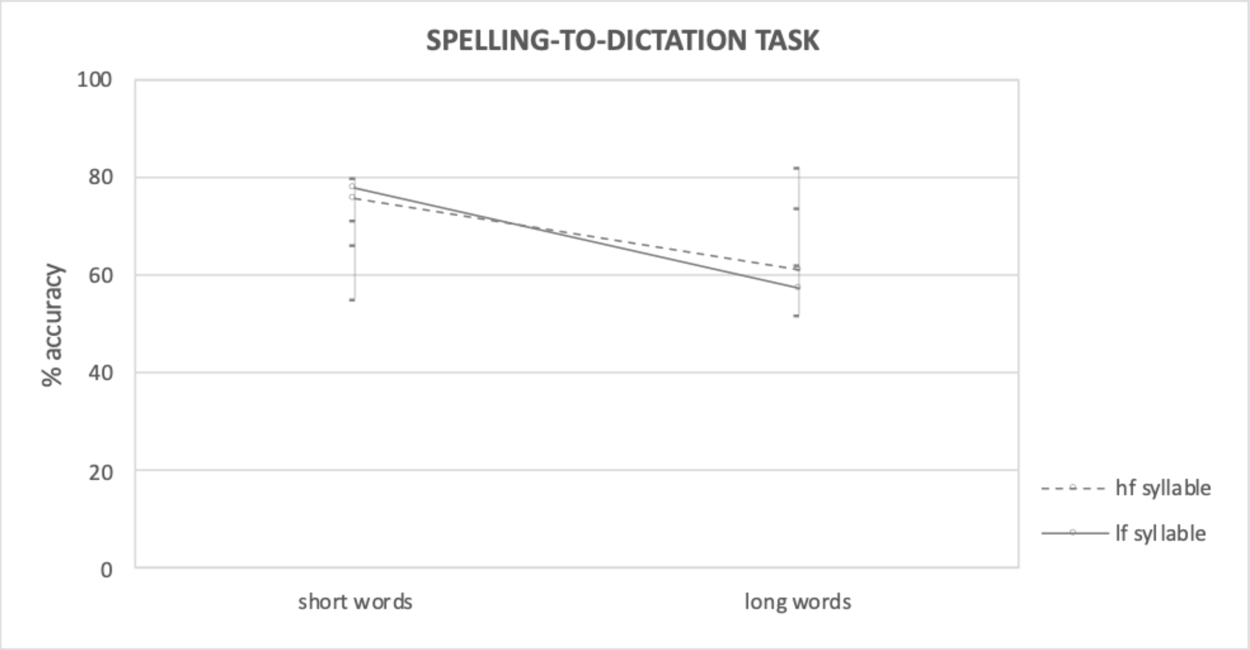

Supplement: S2 Fig — hf = high frequency syllable; lf = low frequency syllable. (PDF) [file pone.0277700.s003.pdf]
