# Supplementary material for: Spelling acquisition in a consistent orthography: The facilitatory effect of syllable frequency in novice spellers
Source: PLoS One. 2022 Nov 14;17(11):e0277700. doi: 10.1371/journal.pone.0277700 (PMC9662710; doi:10.1371/journal.pone.0277700)
Supplement: S1 Table — (PDF) [file pone.0277700.s005.pdf]

S1 Table. Mean percentage of accuracy (and Standard Error) of spelling short and long words, with high and low syllable frequency at T1 (January) and T2 (May).

		T1		T2	
Word Length	Syllable Frequency	Mean	Std.Err.	Mean	Std.Err.
Short	High	72.38	2.73	79.10	3.57
Short	Low	76.93	2.52	78.92	3.29
Long	High	54.18	3.45	67.75	4.50
Long	Low	46.88	3.01	67.39	3.93
